# Developmental Expression and Hypoxic Induction of Hypoxia Inducible Transcription Factors in the Zebrafish

**DOI:** 10.1371/journal.pone.0128938

**Published:** 2015-06-08

**Authors:** Louise Köblitz, Birgit Fiechtner, Katharina Baus, Rebecca Lussnig, Bernd Pelster

**Affiliations:** 1 Institut für Zoologie, Universität Innsbruck, Technikerstr. 25, Austria; 2 Center for Molecular Biosciences, University of Innsbruck, Innsbruck, Austria; 3 Pharmazeutische Biotechnologie, Biberach, Germany; 4 Oncotyrol, Innsbruck, Austria; Institut National de la Recherche Agronomique (INRA), FRANCE

## Abstract

The hypoxia inducible transcription factor (HIF) has been shown to coordinate the hypoxic response of vertebrates and is expressed in three different isoforms, HIF-1α, HIF-2α and HIF-3α. Knock down of either *Hif-1α* or *Hif-2α* in mice results in lethality in embryonic or perinatal stages, suggesting that this transcription factor is not only controlling the hypoxic response, but is also involved in developmental phenomena. In the translucent zebrafish embryo the performance of the cardiovascular system is not essential for early development, therefore this study was designed to analyze the expression of the three Hif-isoforms during zebrafish development and to test the hypoxic inducibility of these transcription factors. To complement the existing zfHif-1α antibody we expressed the whole zfHif-2α protein and used it for immunization and antibody generation. Similarly, fragments of the zfHif-3α protein were used for immunization and generation of a zfHif-3α specific antibody. To demonstrate presence of the Hif-isoforms during development [between 1 day post fertilization (1 dpf) and 9 dpf] affinity-purified antibodies were used. Hif-1α protein was present under normoxic conditions in all developmental stages, but no significant differences between the different developmental stages could be detected. Hif-2α was also present from 1 dpf onwards, but in post hatching stages (between 5 and 9 dpf) the expression level was significantly higher than prior to hatching. Similarly, Hif-3α was expressed from 1 dpf onwards, and the expression level significantly increased until 5 dpf, suggesting that Hif-2α and Hif-3α play a particular role in early development. Hypoxic exposure (oxygen partial pressure = 5 kPa) in turn caused a significant increase in the level of Hif-1α protein even at 1 dpf and in later stages, while neither Hif-2α nor Hif-3α protein level were affected. In these early developmental stages Hif-1α therefore appears to be more important for the coordination of hypoxic responsiveness.

## Introduction

The response of tissues of eukaryotic organisms to reduced oxygen availability is by and large coordinated by hypoxia inducible transcription factors (HIF proteins) [1–5]. HIF proteins are members of the basic helix-loop-helix (bHLH)-Per-Arnt-SIM (PAS) family of transcription factors. By binding of a heterodimeric complex composed of one HIF-α and a HIF-1β compound to hypoxia responsive elements (HRE’s) in the control region of hypoxia responsive genes more than 100 downstream genes are controlled in their transcriptional activity [6]. For most vertebrates three different HIF-α isoforms, HIF-1α, HIF-2α and HIF-3α, and one HIF-1β protein (initially described as ARNT protein) have been described.

HIF proteins are expressed under normoxic conditions. While the HIF-1β protein appears to be constitutively present, the HIF-α subunits are primarily regulated by post-translational control of protein stability [7]. Under normoxic conditions two specific proline residues within the so-called oxygen dependent degradation domain (ODDD) of HIF-1α or of HIF-2α are hydroxylated, inducing an ubiquitination through interaction with the von Hippel-Lindau tumor suppressor protein (VHL), which is the recognition site of the E3 ubiquitination-ligase complex [2,5,8,9]. Proline hydroxylation is catalyzed by proline hydroxylase domain-containing proteins (PHD), which require oxygen as a cofactor. Thus, under normoxic conditions PHD is active, resulting in a rapid degradation of HIF-α isoforms in the proteasomal pathway. Under hypoxic conditions, however, the lack of oxygen inhibits PHD activity and HIF-α proteins accumulate, dimerize with HIF-β, enter the nucleus and act as a transcription factor controlling hypoxia responsive genes.

In addition to the control and coordination of the hypoxic response, HIF proteins have a significant impact on the development and differentiation of organs and tissues. *Hif-1*α or *Hif-2*α knock-out mice die in the embryonic or perinatal stage with different developmental defects [10–12]. Malformations of the neural tube and various cardiovascular defects suggest that HIF proteins are involved in the differentiation of neuronal and cardiovascular tissues. Some evidence also connects HIF protein to the formation of cartilage [13,14]. The phenotype of *Hif*-2α ^-/-^ mice apparently is dependent on the background and ranges from embryonic lethality due to vascular defects or mid-gestational bradycardia, or perinatal death because of respiratory distress [10,15,16].

Mammalian development within the uterus makes a detailed analysis of the specific role of HIF protein during development quite difficult. Accordingly, the developmental role of HIF protein has also been addressed in Xenopus or in zebrafish with free living embryonic and larval stages, which due to their transparency even allow for a microscopic analysis of developmental processes. Using *hif-1*α promoter constructs specific temporal and spatial differences in the *hif-1*α activity have been described for *Xenopus laevis* development [17]. In these studies an elevated expression of *hif-1*α mRNA was reported for the nervous system and axial structures, supporting the hypothesis that HIF-1α might be important for proper development and differentiation of the nervous system. Analysis of *hif-1*α and *hif-2*α mRNA expression in the zebrafish using in situ hybridization revealed expression of both isoforms throughout development with distinct temporal and spatial patterns, suggesting in part overlapping, but also non-redundant functions [18]. Presence of *hif-3*α mRNA as early as 4h after fertilization was first reported by Zhang et al., and it was monitored until 4 dpf [19].

A detailed analysis of the gene structure of Hif–α proteins revealed the presence of two paralogs for each of the three Hif- α isoforms in zebrafish. Whole genome duplication in teleosts resulted in paralogous gene pairs of all Hif-α proteins (*hif-1A*,*B*, *hif-2A*,*B*, *hif-3A*,*B*), which, in contrast to most teleost species, have been retained in cyprinid fish, and thus in zebrafish [20,21]. An expression analysis demonstrated that the mRNA of all paralogs except for *hif-3B* can be detected as early as 8 hours after fertilization and in subsequent development. *hif-3B* was first detected 24h after fertilization. Furthermore, the expression of all paralogs except for *hif-2B* was modified under hypoxic conditions, and the mRNA concentration typically was lower in 2 dpf embryos as compared to normoxic animals [20,21]. The authors speculate that the evolutionary retention of two paralogs of each of the three Hif-α proteins allows for a functional divergence of the paralogs. Unfortunately, however, information about the presence of Hif-α proteins in early developmental stages of the zebrafish is fragmentary and scarce.

Due to the post-translational regulation of HIF-α protein stability, however, knowledge of the HIF protein expression is required to assess the possible contribution of this transcription factor to developmental processes. Therefore we generated specific antibodies against zebrafish Hif-1α, Hif-2α and Hif-3α in order to assess the expression of all three isoforms during development and their expression changes during hypoxic exposure. The results revealed expression of all three isoforms throughout development with characteristic developmental patterns. Hypoxic exposure caused a significant elevation in the level of Hif-1α protein even at 1 day post fertilization (dpf) and in later stages, while neither Hif-2α nor Hif-3α protein level were significantly affected.

## Materials and Methods

### Animals and experimental design

The experiments were performed with zebrafish larvae (*Danio rerio*) obtained from our breeding colony. Larvae were kept at the experimental temperature of 25°C with a 14:10 light dark photoperiod. Adult stocks were continuously reconditioned by new fish from a local supplier to avoid inbreeding. Animal experiments were performed according to our animal ethics permission GZ 66.008/4-BrGT/2004 of the Austrian Federal Ministry for Education, Arts and Culture.

Fertilized eggs were kept in small breeding tanks.

Control groups were raised in normoxia (PO_2_ ~ 20 kPa). A first series of hypoxic incubations started with larvae after hatching. Hypoxic treatment groups were incubated at a PO_2_ of 5 kPa by the controlled inflow of an air-nitrogen gas mixture (PreSense, Regensburg, Germany), starting at 3 dpf. In previous studies focusing on the development of the cardio-vascular system we had shown that this level of hypoxia after hatching does not interfere with development [22], and we intended to analyze the expression of Hif-proteins under conditions that did not result in developmental retardation. Samples were taken daily between one and 9 dpf, 8 h after lights on. In a previous study we could demonstrate that with the light regime 14/10 L/D at this time point a high level of Hif-1α protein can be expected [23].

In a second set of experiments we then analyzed whether hypoxia results in a stabilization of Hif-proteins already in embryonic stages. Embryos were exposed to hypoxia for 4 h at 1, 2 and 3 dpf. Prolonged incubation of embryos at a PO_2_ of 5 kPa resulted in delayed hatching or even in an increase in mortality. Therefore the incubation time was limited to 4h. In order to match the sampling time at 8 h after lights on the 4 h hypoxic exposure started 4 h after lights on, i.e. at 28 h after fertilization (1 dpf), at 52 hpf (2 dpf) or at 76 hpf (3 dpf).

### Plasmids, antibodies, fusion protein expression/purification

To demonstrate presence of zfHif-1α protein a zfHif-1α specific antibody previously described has been used [24]. A zebrafish Hif-2α specific antibody was generated using a similar procedure. Briefly, full-length zebrafish Hif-2α was amplified from larval zebrafish cDNA using the following primers: forward primer 5'-CGG GGG AAT TCA TGA CAG CTG AGA AAGAG-3'; reverse primer 5'-CTC GAG CGG TCT AAG TCG CCT GGT C-3'. The PCR product was cloned into the *Eco*RI/*XhoI* sites of a pGEX-4T-1 vector (GE Healthcare). The fusion protein was expressed in BL21(DE3) cells (Invitrogen) as described previously [24]. For expression of fusion proteins, *E*.*Coli* BL21(DE3) where transformed with zfHif-2α pGEX-4T-1. Bacteria were grown in LB/Amp medium at 37°C at 220 rpm to an optical density of approx. 0.8 at 600nm. Bacteria were cooled to 20°C while shaking at 220 rpm (30min) before induction of fusion protein expression was started by addition of 0.1mM IPTG. Induction of fusion protein expression was performed for 2 hrs at 20°C. Bacterial cultures were centrifuged at 4,000 rpm in a Heraeus Labofuge 400R (#8179 rotor) for 20 min (4°C) to precipitate the cells.

Subsequently, the bacterial cell pellets were lysed by the addition of lysozyme and subsequent ultrasonification on ice using the Ultrasonic processor UP200S (Dr. Hielscher, 6 cycles, 10 seconds each at 70% of maximal power). The bacterial lysate was centrifuged 30 min at 4°C (Eppendorf Centrifuge 5415R) to remove the insoluble bacterial debris. The cleared lysate was incubated with washed glutathione beads (Glutathione Sepharose Fast Flow, GE Healthcare) for 1hr at 4°C. Bacterial fusion proteins were solubilized by addition of 10mM reduced glutathione in 10mM Tris, pH 8.5 (10 min overhead rotation, 4°C). Buffer exchange and concentration was achieved using Amicon Ultra-15 Centrifugal Filter Units (EMD Millipore Corporation).

Protein concentrations were determined with NanoDrop 2000c (Thermo Scientific).

The expressed GST-tagged full-length zebrafish Hif-2α (amino acids 1–832) was used for immunization of rabbits to obtain a polyclonal antibody. The fourth immune bleed (obtained 70 days after initial immunization) was taken for affinity purification against the full length bacterial expressed GST-tagged zebrafish Hif-2α protein (Eurogentec, Seraing, Belgium). The antibody specifically detects zfHif-2α in zebrafish larvae at the expected running position of 93 kDa.

A zfHif-3α specific antibody was generated by using zfHif-3α-specific peptides corresponding to amino acids 76–90 [csqteksetptdgfyq] and amino acids 566–580 [csdgsdefepppqkrs] for the immunization of rabbits. A similar strategy has successfully been used to generate a Hif-4α specific antibody for grass carp [25], which corresponds to zfHif-3α [24,26]. The peptides were custom produced and the antibodies affinity purified by Delphi Genetics (Belgium).

### Protein extraction

Zebrafish embryos were dechorionated and deyolked by pipetting in PBS and shock-frozen in 2x Laemmli sample buffer (BioRad). Samples where boiled at 95°C for 30 min and dispersed by frequent and rapid pipetting. Undissolved proteins and pigment was separated by centrifugation 10 min, 16000 rpm (Eppendorf Centrifuge 5415R). Protein concentration was determined by measurement of total protein absorption at 280nm with the NanoDrop 2000c (Thermo Scientific) in triplicates.

### Western Blotting

Western blotting was performed as described previously [24]. 150μg of protein per sample were loaded to 12% precast SDS-PAGE gels (18 well Criterion TGX Stain-Free Gels, BioRad, and blotted to PVDF or to Nitrocellulose membranes, respectively. Equal loading was verified by UV exposure in the Chemidoc XRS+ (BioRad). Unspecific protein binding sites were blocked by preincubating the membranes in Tris-buffered saline containing 5% skim milk powder and 0.1% Tween 20 (blocking buffer) for 45 min at room temperature. Primary polyclonal Hif antibodies were diluted in blocking buffer either 1:1000 (Hif-1α) or 1:2000 (Hif-2α /3α). Incubation with primary antibody was performed overnight at 4°C. Binding of the primary antibody was detected with a secondary antibody conjugated to horseradish peroxidase (Abcam, Cambridge, UK) diluted 1:10,000 in blocking buffer for 1 h at room temperature by enhanced chemiluminescence detection (Amersham ECL Select Western Blotting Detection Reagent). Band density was analyzed with Image Lab 4.1 software (BioRad).

As discussed previously [24], no gene has been described yet that may reliably serve as housekeeping gene in developing zebrafish larvae, especially if development is combined with environmental stress conditions like hypoxic exposure. Therefore, protein concentration was accurately determined in triplicates using a NanoDrop 2000c (Thermo Scientific). In addition, Western blot loading was checked by UV exposure of the BioRad Stain free gels. Comparing overall intensity of protein staining of the different lanes demonstrated equal loading of all samples.

### Data analysis and statistics

Western blot data were analyzed using two-way ANOVA followed by Holm-Sidak multiple comparison procedures using SigmaStat 12.0 for the comparison of different developmental stages and testing the influence of PO_2_ on Hif-protein concentration. Data are shown as mean ± SEM. Significant differences were accepted for P< 0.05 and are indicated by letters or asterisks in the respective figures.

## Results

**Fig 1:**
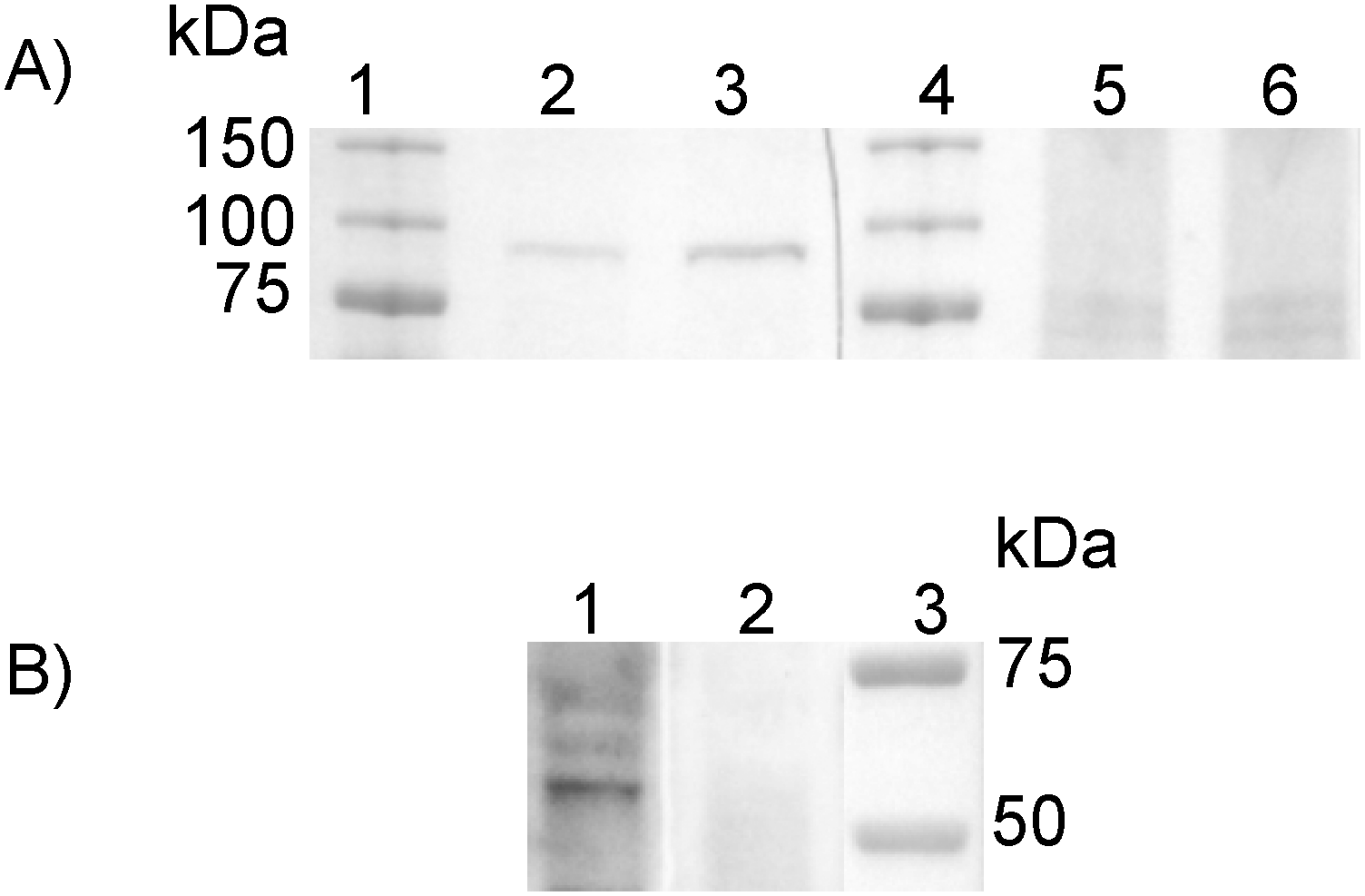
Specificity test for the zfHif-2α and the zfHif-3α antibody. A,150 μg total protein of normoxic (lane 2) and hypoxic (lane 3) zebrafish larvae at 5 dpf have been subjected to SDS electrophoresis. Incubation with the zfHif-2α specific antibody results in a band at 93 kD. Preincubation of the antibody with recombinant zfHif-2α protein eliminates this band (lane 5, normoxic, lane 6, hypoxic sample). Lane 1 and 4 show the marker. B, 150 μg total protein of hypoxic (lane 1) zebrafish embryo at 3 dpf have been subjected to SDS electrophoresis. Incubation with the zfHif-3α specific antibody results in a band at 69 kD. Preincubation of the antibody with recombinant zfHif-3α protein eliminates this band (lane 2). Lane 3 shows the marker.

While the specific antibody directed against zfHif-1α protein has been used in previous studies [23,24], for this study additional antibodies had to be generated against zfHif-2α and zfHif-3α protein. For the generation of a specific zfHif-2α antibody the expressed Hif-2α protein was used as an antigen, and specificity of the obtained antibody was validated by successfully blocking the signal with the recombinant zfHif-2α protein (Fig 1A). Western-blot using tissue from normoxic 5 dpf larvae showed a distinct band at 93 kDa, which was intensified in the hypoxic incubated larvae (24 h; PO_2_ = 5 kPa). After blocking with recombinant Hif-2α protein, this band was no longer detectable. No band was detectable at 86 kDa or 69 kDa, the molecular weight of zfHif-1α and zfHif-3α, respectively. Similarly, specificity of the Hif-3α antibody was validated using the peptides used as an antigen to block the signal or the recombinant zfHif-3α protein. Tissue from hypoxic larvae at 3 dpf showed a band at 69 kDa, which was successfully blocked after preincubation with the Hif-3α peptides (data not shown) or with the recombinant zfHif-3α protein (Fig 1B).

**Fig 2:**
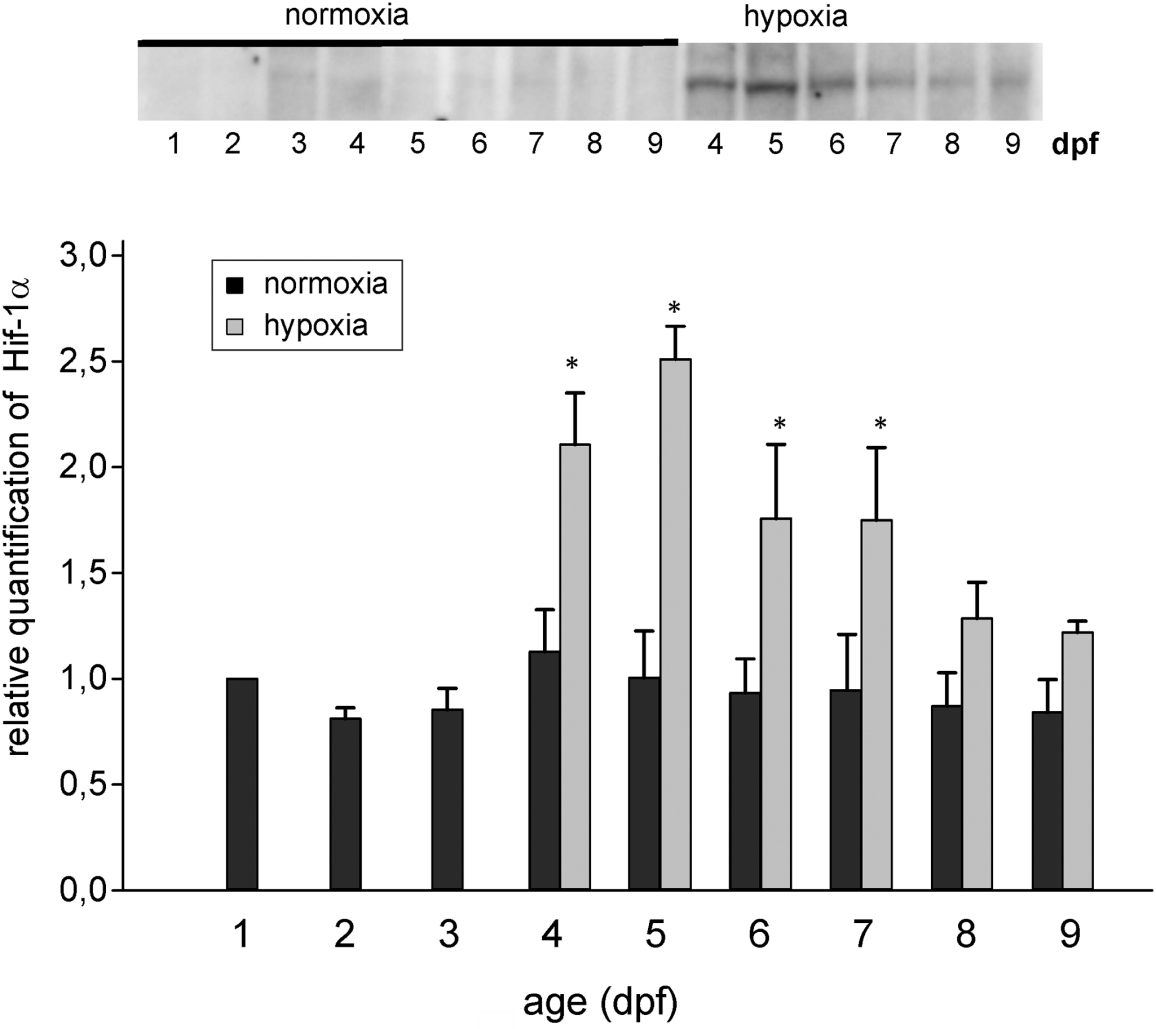
Western blot and relative quantification of total zebrafish protein between 1 and 9 dpf under normoxic conditions, and of larvae incubated under hypoxic conditions (PO_2_ = 5 kPa) starting at 3 dpf tested with the zfHif-1α specific antibody. * indicate significant differences between hypoxic and normoxic samples (p<0.05, n = 5).

Western blot using the zfHif-1α specific antibody showed a faint band at 86kDa at 1 day post fertilization, indicating that Hif-1α protein is present in the first stages of embryonic development of the zebrafish. During the subsequent development until 9 dpf the band was present with similar intensity (Fig 2). In this first series of experiments larvae were exposed to reduced oxygen concentration (5 kPa), from 3 dpf onwards, and after 24h of hypoxia (4 dpf) Hif-1α protein was significantly elevated compared to normoxic control levels (Fig 2). In the hypoxic group Hif-1α protein remained significantly elevated compared to the control group until 7 dpf. It was also elevated at 8 and 9 dpf, i.e. after 5 and 6 days of hypoxia, but this difference was no longer significant.

**Fig 3:**
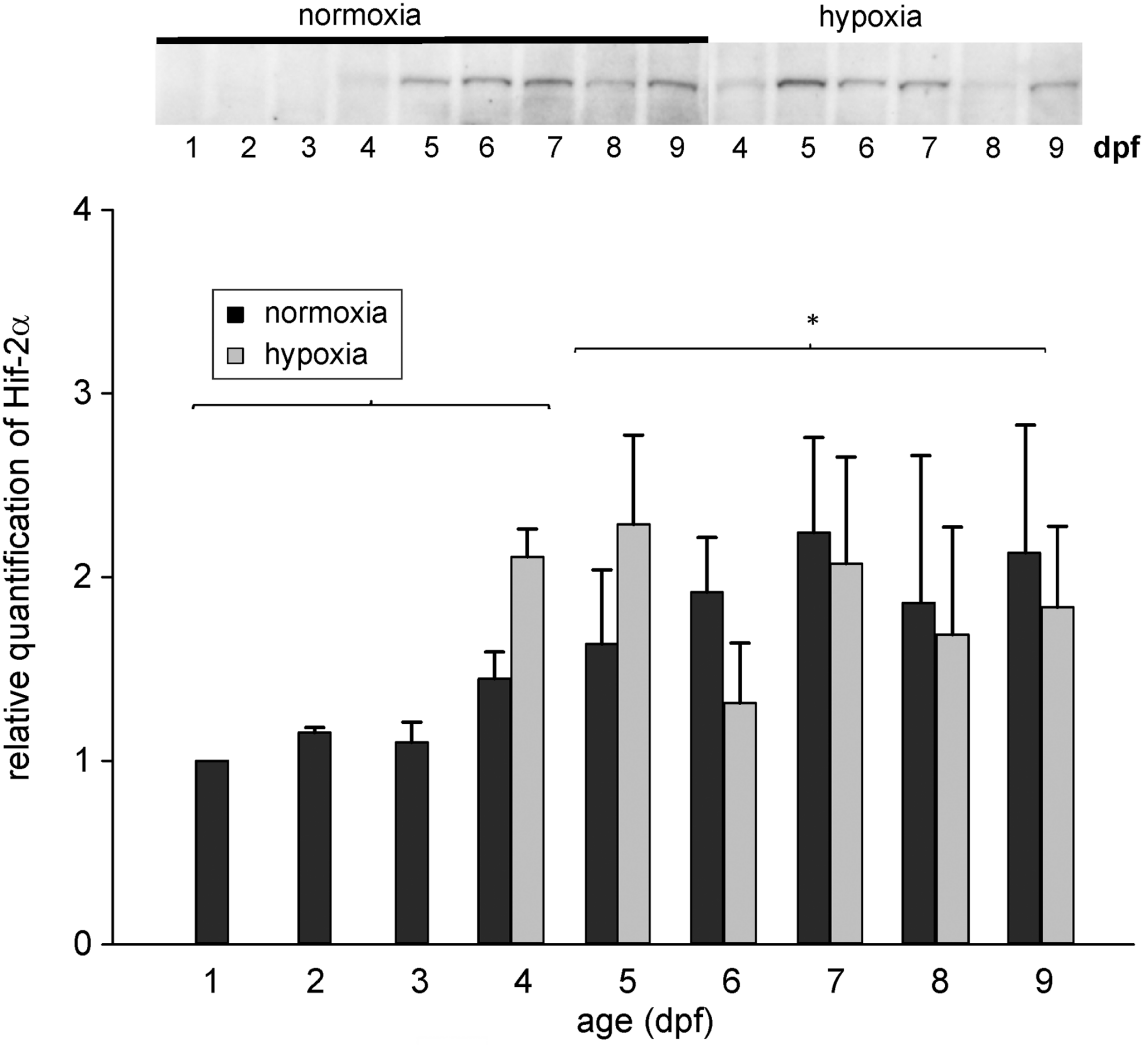
Western blot and relative quantification of total zebrafish protein between 1 and 9 dpf under normoxic conditions, and of larvae incubated under hypoxic conditions (PO_2_ = 5 kPa) starting at 3 dpf tested with the zfHif-2α specific antibody. * indicates significant differences between normoxic samples of day 1 to 4 and 5 to 9 (p<0.05, n = 5).

Hif-2α protein was also present throughout development between 1 and 9 dpf with a band at 93 kDa. In contrast to Hif-1α, which remained constant with development, the level of Hif-2α increased during development, and between 5 and 9 dpf it was significantly higher than between 1 and 4 dpf (Fig 3). In larvae exposed to hypoxic conditions starting at 3 dpf the level of Hif-2α protein remained similar to the level measured in normoxic control larvae of the same developmental stage (Fig 3). Accordingly, hypoxic conditions did not result in an elevated Hif-2α protein content.

**Fig 4:**
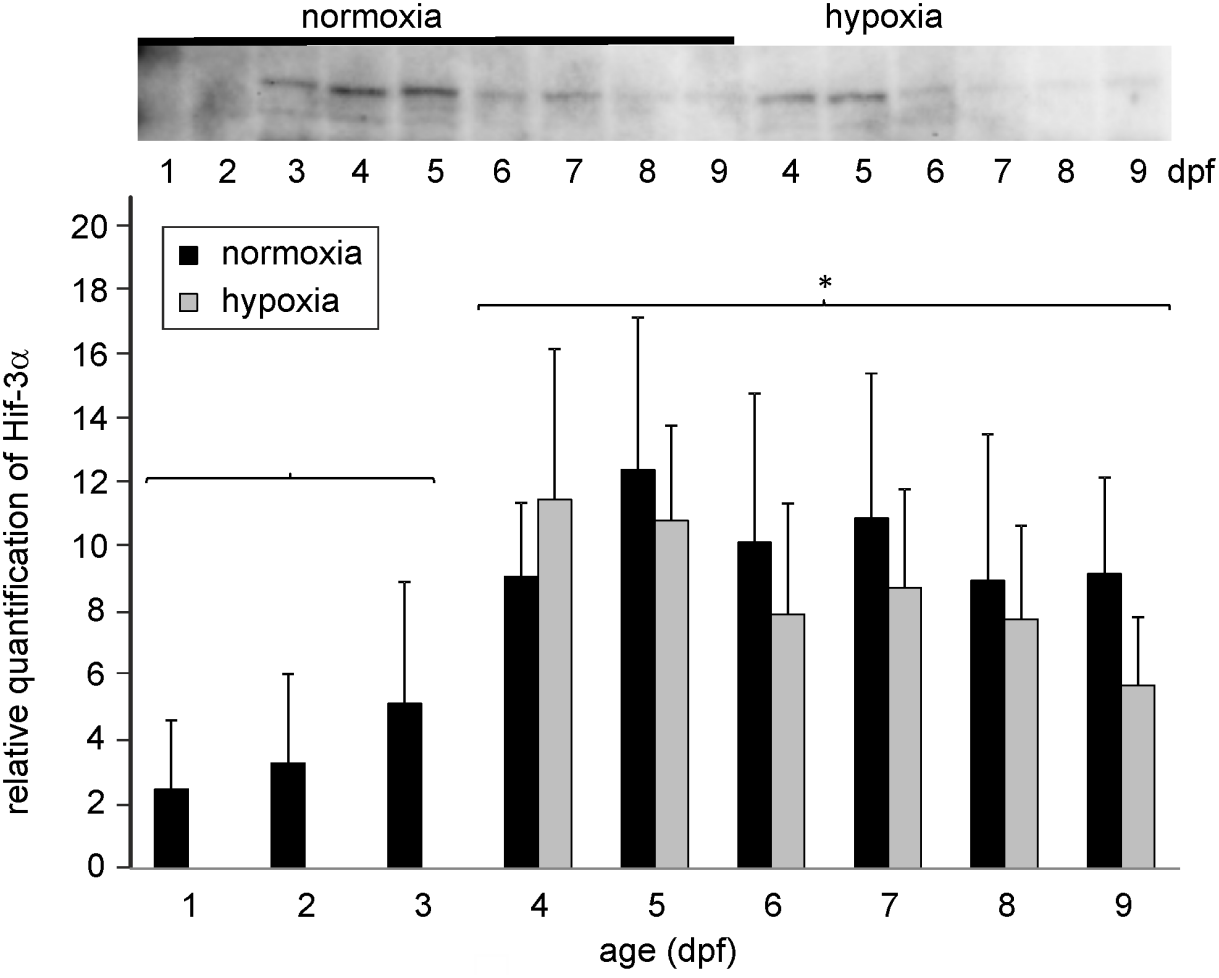
Western blot and relative quantification of total zebrafish protein between 1 and 9 dpf under normoxic conditions, and of larvae incubated under hypoxic conditions (PO_2_ = 5 kPa) starting at 3 dpf tested with the zfHif-1α specific antibody. * indicates significant differences between normoxic samples of day 1 to 3 and 4 to 9 (p<0.05, n = 6).

Fig 4 summarizes the changes in the expression of Hif-3α protein during development between 1 and 9 dpf and under hypoxic conditions. Hif-3α protein was also present in all stages analyzed, starting with 1 dpf. Similar to Hif-2α protein the concentration of Hif-3α protein increased during development and reached the highest value at 5 dpf. Between 4 and 9 dpf the concentration of Hif-3α protein was significantly higher during the first 3 days of development. Hypoxic conditions during development between 3 and 9 dpf did not result in significant differences in the concentration of Hif-3α protein (Fig 4) in hypoxia incubated larvae as compared to normoxic larvae of the same developmental stage.

**Fig 5:**
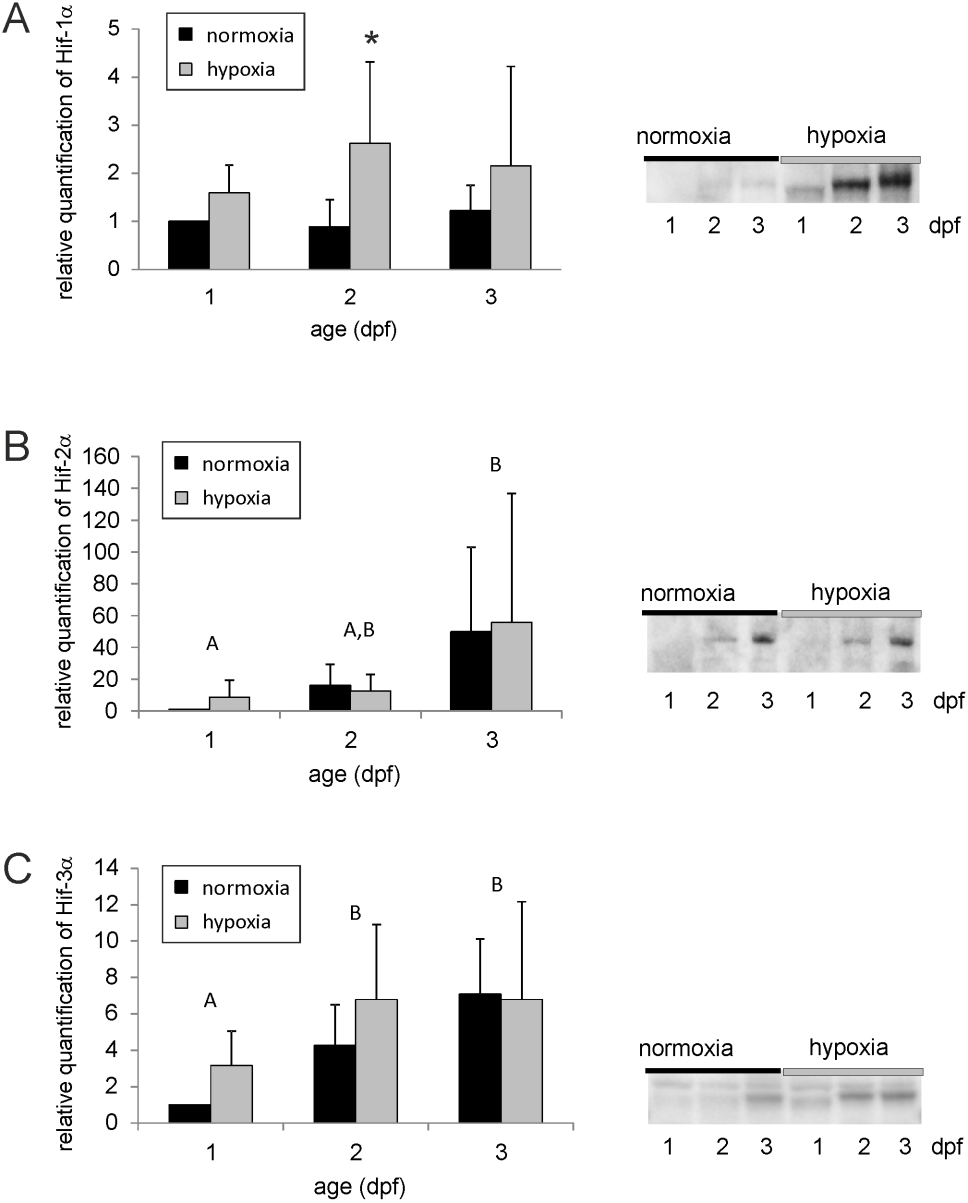
Western blot and relative quantification of zfHif-1α protein (A), of zfHif-2α protein (B) and of zfHif-3α protein (C) of normoxic zebrafish embryos and of embryos incubated under hypoxic conditions (PO_2_ = 5 kPa) for 4 hours starting at 28 h after fertilization (1 dpf), 52 h after fertilization (2 dpf) or 76 h after fertilization (3 dpf) (mean ± SEM). All samples were taken at the end of the respective hypoxic incubation. Different capital letters indicate significant differences between different sampling days (= developmental differences); * indicates significant differences between control and hypoxic groups at the same day. (p<0.05, n = 4).

In order to test the idea that Hif-α proteins may be stabilized even in the earliest developmental stages we incubated embryos at 28 h, 52 h or 76 h after fertilization for 4 hrs under reduced oxygen availability (PO_2_ = 5 kPa). Analysis of Hif-α protein expression revealed an elevation in Hif-1α protein concentration even at this early stage (Fig 5A). Embryos incubated in hypoxia starting at 28 h after fertilization showed an increase in Hif-1α protein after 4 h of hypoxia, and a 4 h hypoxic incubation, started at 52 h after fertilization, resulted in a more than two-fold increase in Hif-1α protein concentration, which was highly significant. At 3 dpf the Hif-1α protein concentration was again elevated following hypoxia, but this increase was no longer significant. In contrast to Hif-1α, Hif-2α and Hif-3α concentration remained unchanged in response to hypoxic conditions (Fig 5B and 5C). Two-way Anova revealed a significantly elevated Hif-2α protein concentration at 3dpf as compared to 1 dpf, and Hif-3α protein concentration was significantly elevated at 2 and 3 dpf as compared to 1 dpf (p<0.05).

## Discussion

### Hif proteins in development

Presence of the mRNA of *hif-1*α, *hif-2*α and *hif-3*α in early developmental stages of the zebrafish has been demonstrated previously [18–21]. Due to the post-translational regulation of HIF-α protein stability and the repeatedly observed discrepancies between mRNA and protein concentrations [27,28] in cells and tissues, however, information about the expressed protein is essential to elucidate the possible physiological importance of Hif-transcription factors. Our results show for the first time that at least one paralog of all three Hif- α proteins is present in the developing zebrafish embryo 24h after fertilization, and remains detectable until 9 dpf. Following the analysis of Rytkönen et al. the isoforms analyzed in this study are Hif-1B, Hif-2A and Hif-3A, and these also appear to be isoforms that have been analyzed in previous studies [21]. Taking into account, however, that for *hif-2A* and *hif-2B* mainly transcriptional divergence has been reported [21] and that we used the full length expressed protein for immunization we cannot exclude that our polyclonal antibodies do not separate between Hif-2A and Hif-2B, assuming that both transcripts are indeed translated in the larvae.

In contrast to Hif-1α (Hif-1B), which remained at the lower level of detectability throughout development, Hif-2α (Hif-2A) and Hif-3α (Hif-3A) expression was significantly higher in the last part of development as compared to the embryonic phase (1dpf-3dpf). For the mRNA of Hif-1B an almost 5-fold increase was reported between 8 h and 48 h post fertilization under normoxic conditions [21], while in an earlier study it remained constant between zygote and 7 dpf [20]. There is no explanation for this discrepancy, but if we take the latter study it appears that neither the mRNA nor the protein of Hif-1B show significant changes during early development. The mRNA of Hif-2A, in contrast, showed a stable expression level until hatching and then increased about 3-fold [20,21]. Our protein data also show an increase with development, stabilizing between 5 dpf and 9 dpf. For Hif-3A mRNA Rytkönen et al. [21] reported an increase between 12 h and 48 h of development. This would be consistent with the increase in the protein level observed in embryonic stages in our study (Fig 5). Taken together it appears as if the changes in the protein level of the three Hif isoforms observed in our study during early development are more or less consistent with the observed changes in the mRNA concentration of the respective isoform.

Knock out of *Hif-1*α in mice resulted in embryonic lethality at E9-E11 with defects in the cardiovascular system, in angiogenesis and neuronal tissue [11,29], demonstrating that HIF-1α is indispensable during early development. If this is similar in zebrafish, our results suggest that the basic level of zfHif-1α is sufficient to assure proper development of the tissues, a particular accumulation of the protein is not required.

This situation appears to be different for Hif-2α and Hif-3α, which are significantly elevated between 5dpf and 9 dpf, compared to the earlier developmental stages. Similar to *Hif-1*α k.o., in mice *Hif-2*α k.o is lethal at the embryonic or perinatal stage, but with a different phenotype. *Hif-2*α k.o. mice die with defective surfactant production, an impaired lung function and vascular defects [10,15,16]. The higher Hif-2α protein concentration observed beyond 5 dpf as compared to the first days of embryonic development suggests that this transcription factor may be of increased importance during later phases of tissue differentiation and growth. The possible importance of this protein for proper development of the vascular system may be similar in mice and zebrafish, but its role for surfactant production in a lung-less fish is not obvious at first glance. Recent molecular data, however, has shown that the swimbladder is homologues to the mammalian lung [30,31], and, similar to the lung, swimbladder inflation does also require surfactant [32,33]. It therefore may be speculated that Hif-2α may play a role in swimbladder differentiation and development.

The role of Hif-3α during development has not yet been considered in detail. The elevated concentration observed between 4 dpf and 9 dpf as compared to 1dpf to 3 dpf suggests that it is involved in development, but no particular function has yet been ascribed to this protein. The traditional view assumes that HIF-3α simply is a competitive inhibitor of HIF-1α and HIF-2α because it is a more or less truncated version of the HIF-α proteins that lacks the C-terminal transactivation domain, and the main function of this domain is to recruit cofactors like p300/CBP, which in turn are crucial for the activation of hypoxia responsive genes [34,35]. For some of the different HIF-3α isoforms the inhibitory activity has indeed been demonstrated, apparently competing with HIF-1α and HIF-2α for HRE binding sites [36–40]. Recent studies, however, report that HIF-3α is able to drive transcription by itself and therefore cannot only be considered to be a competitive inhibitor [36,41]. Zhang et al. [42] in an elegant study using zebrafish as a model organism identified genes specifically under the control of Hif-3 α while others appeared to be under the control of Hif-1α as well as of Hif-3 α. While in our study the level of Hif-3α protein was elevated between 4 and 9 dpf as compared to earlier stages of development, the level of Hif-1α protein remained low in these stages of development. Taking into account that Hif-3α can drive transcription [42], this suggests that in zebrafish development Hif-3α may play a specific role as a transcription factor, exceeding its possible function as a competitive inhibitor of the other two Hif-α isoforms.

### Hif protein accumulation during hypoxic exposure

The post-translational regulation of HIF-α protein stability assures accumulation of these transcription factors under hypoxic conditions [2,5,8,9]. A comparison of the changes in protein expression observed in our study under hypoxic conditions with the data available on mRNA expression under these conditions reveals that changes in mRNA expression are not necessarily indicative of changes in protein concentration. While in a previous study using zebrafish embryos a change in the mRNA of Hif-1α under hypoxic conditions (5% oxygen) has been reported [43], in the recent study of Rytkönen et al. Hif-1B mRNA concentration remained at a similar level in normoxic and hypoxic embryos between 24 h and 48 h after fertilization [21]. Our results show that the protein level was significantly increased in hypoxic animals, demonstrating a stabilization of the protein under these conditions. A discrepancy between Hif-1α (Hif-1B) mRNA and protein concentration has also been observed in an earlier study. Zebrafish larvae incubated under hypoxic conditions (PO_2_ = 5 kPa, starting at 9 dpf) showed an increase in Hif-1α mRNA at 14 and 15 dpf, while the protein level increased during the first days of hypoxia, but was back to normoxic control levels at 15 dpf [24]. For Hif-2A and Hif-3A, compared to normoxic embryos in hypoxic embryos a slight decrease in the mRNA concentration was observed between 24 h and 48 h after fertilization [21], while our results showed no difference for Hif-2A and of Hif-3A protein concentration between normoxic and hypoxic animals. Using much lower PO_2_ values (~ 2 kPa) an increase in Hif-3A mRNA and protein has been reported for zebrafish embryos incubated in hypoxia for 24 h starting at 6 h after fertilization [19]. Taken together it appears that in particular under hypoxic conditions changes in mRNA concentrations of Hif-proteins are not indicative for concomitant changes in Hif-protein concentration.

In previous studies accumulation of Hif-1α in situations of reduced oxygen availability has been demonstrated for zebrafish during the first day of development [44], and between 9 and 15 dpf [24]. Our results confirm the hypoxic responsiveness for Hif-1α, but neither Hif-2α nor Hif-3α showed a significant increase in concentration during hypoxic exposure. This was surprising, because the post-translational regulation should be similar for all three proteins [45]. A possible explanation for this observation would be that Hif-2α and Hif-3α are only stabilized at lower PO_2_ values. In addition, the different binding characteristics of the specific antibodies do not allow for relative quantification of the three Hif-proteins, but conceivably Hif-2α and Hif-3 α protein overall are present at a lower concentration than Hif-1α, and a possible tissue specific increase in protein concentration could be obscured because for the Western blot analysis whole animal protein had to be collected. In contrast to our observation Zhang et al. [19] reported a significant increase in Hif-3α protein in zebrafish embryos raised in hypoxia starting 6 h after fertilization. In their study a PO_2_ of below 2 kPa (9% of aerial PO_2_) was used, which is much lower than the values used in our present study, and because closed respirometry has been used in their study the actual PO_2_ may even have been lower by the end of the experiment. This supports the hypothesis that in these early embryos Hif-3α stabilisation occurs only at very low PO_2_ levels. According to our observations, however, a normal development of zebrafish embryos is not possible at this low level of oxygen. In a previous experiment we incubated zebrafish larvae under hypoxic conditions (PO_2_ = 5 kPa) starting at 9 dpf, and we observed an increase in Hif-3α protein after three days of hypoxia, i.e. at 12 dpf [24]. This increase was transient, and at 15 dpf the Hif-3α concentration in hypoxic larvae was no longer higher than in control larvae raised at normoxic PO_2_.

Taken together this observation suggests that focusing on the Hif-family of transcription factors Hif-1α may play a dominating role for hypoxic adaptation in these early stages of zebrafish development. Furthermore, the post-translational regulation of Hif-1α stability is functioning and in place in the earliest embryonic stages. The significant increase in cardiac activity, a classical down-stream response of Hif-signaling, appears to be slightly delayed [22] compared to this immediate response of the Hif-1α protein. The signaling pathway to increase cardiac activity comprises several steps, of which the accumulation of Hif-proteins is the very first one. At this stage the stimulation of the heart and possible additional vascular responsiveness most likely is achieved by hormonal action, due to the slightly delayed onset of the functioning of the autonomous nervous system [46,47]. Activation of an endocrine organ, hormone production and release and the distribution to the target organs takes time and thus may contribute to the delayed response.

### Perspectives

Fish are the largest and most diverse group of vertebrates, and due to the low oxygen solubility in water frequently exposed to conditions of low oxygen availability. A number of studies using different species of fish demonstrated that fish extensively explored und make use of the Hif-signaling pathway to cope with hypoxic conditions. Accordingly, fish have successfully been used as model species to entangle the details and enigmas of the HIF-signaling pathway. The present study suggests that in fish Hif-proteins—like in mammals—may also be involved in embryonic and larval tissue differentiation and organ formation. Due to the transparency of tissues and the development in the water fish therefore are ideal to entangle possible isoform specific functions of Hif-1α, Hif-2α and Hif-3α in development and organ differentiation. A detailed analysis of the tissue specific distribution of the three proteins in different stages of development and the effect of a knock down or an over-expression will significantly advance our knowledge on the role of these transcription factors during development.
